# Human osteogenic differentiation in Space: proteomic and epigenetic clues to better understand osteoporosis

**DOI:** 10.1038/s41598-019-44593-6

**Published:** 2019-06-06

**Authors:** Alessandra Gambacurta, Giulia Merlini, Cristina Ruggiero, Giacomo Diedenhofen, Natalia Battista, Monica Bari, Michele Balsamo, Sara Piccirillo, Giovanni Valentini, Gabriele Mascetti, Mauro Maccarrone

**Affiliations:** 1 Department of Experimental Medicine, Tor Vergata University of Rome, Rome, Italy; 2 NAST Centre for Nanoscience, Tor Vergata University of Rome, Rome, Italy; 3 Faculty of Bioscience and Technology for Food, Agriculture and Environment, University of Teramo, Teramo, Italy; 4 Kayser Italia S.r.l., Livorno, Italy; 5 Italian Space Agency, Rome, Italy; 6 Department of Medicine, Campus Bio-Medico University of Rome, Rome, Italy; 7 European Center for Brain Research, IRCCS Santa Lucia Foundation, Rome, Italy

**Keywords:** Diagnostic markers, Proteomics

## Abstract

In the frame of the VITA mission of the Italian Space Agency (ASI), we addressed the problem of Space osteoporosis by using human blood-derived stem cells (BDSCs) as a suitable osteogenic differentiation model. In particular, we investigated proteomic and epigenetic changes in BDSCs during osteoblastic differentiation induced by rapamycin under microgravity conditions. A decrease in the expression of 4 embryonic markers (Sox2, Oct3/4, Nanog and E-cadherin) was found to occur to a larger extent on board the ISS than on Earth, along with an earlier activation of the differentiation process towards the osteogenic lineage. The changes in the expression of 4 transcription factors (Otx2, Snail, GATA4 and Sox17) engaged in osteogenesis supported these findings. We then ascertained whether osteogenic differentiation of BDSCs could depend on epigenetic regulation, and interrogated changes of histone H3 that is crucial in this type of gene control. Indeed, we found that H3K4me3, H3K27me2/3, H3K79me2/3 and H3K9me2/3 residues are engaged in cellular reprogramming that drives gene expression. Overall, we suggest that rapamycin induces transcriptional activation of BDSCs towards osteogenic differentiation, through increased GATA4 and Sox17 that modulate downstream transcription factors (like Runx2), critical for bone formation. Additional studies are warranted to ascertain the possible exploitation of these data to identify new biomarkers and therapeutic targets to treat osteoporosis, not only in Space but also on Earth.

## Introduction

Bone remodeling is an ongoing process, fundamental for adapting bone architecture to growth and repair processes, and for maintaining calcium homeostasis. Bone remodeling depends on the interaction between osteoblastic cells of mesenchymal origin and osteoclastic cells of hematopoietic origin.

Alterations of the equilibrium between bone resorption and bone formation can determine reduced bone density and increased fragility, hallmarks of osteoporosis^1–3^. On Earth, environmental elements (diet, physical activity, endocrine status, smoking, etc.) and genetic predisposition contribute to the onset of osteoporosis^4–6^.

In Space, however, astronauts lose on average more than 1% of bone mass per month and, while progression to osteoporosis is not an issue for short-term flights, it is indeed a handicap to long-term missions such as those on the International Space Station (ISS) and interplanetary flights. The cause of developing osteoporosis in Space is linked to low (micro- to zero-) gravity conditions, with possible contributions of cosmic ray radiation and circadian rhythm alterations^7–9^. In recent years, Space research has focused on osteoporosis in search for possible therapies able to counteract bone mass loss, crucial to prolong future manned Space missions.

Human blood-derived stem cells (BDSCs) are ideal candidates to study bone mass loss that affects astronauts, as these cells are autologous and pluripotent, and are able to differentiate into many different cell types. Of note, their osteogenic differentiation induced by rapamycin in the presence of suitable scaffolds has been widely studied^10–15^. Recently, rapamycin has also been approved for the treatment of bone pain in patients with bone metastases, due to its ability to counterbalance the effects of osteoclasts by promoting osteoblast activity^16,17^.

**Figure 1:**
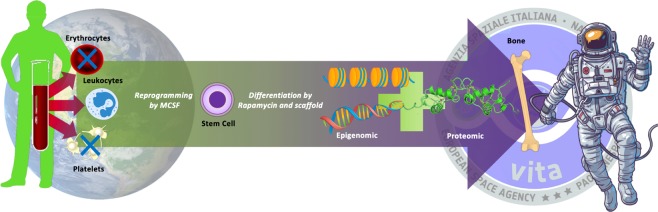
Rationale of the SERiSM project. Rapamycin-driven osteogenesis of human blood-derived stem cells (BDSCs) on board the ISS. MCSF, macrophage colony-stimulating factor.

Recently, we performed the “SERiSM” (Role of the Endocannabinoid System in Reprogramming Human Pluripotent Stem Cells under Microgravity) project, that was selected by the Italian Space Agency (ASI) on a competitive basis, in order to: i) evaluate the osteogenic differentiation of BDSCs under real microgravity, and ii) interrogate the possible modulation of the so-called “endocannabinoid system” (i.e., a complex ensamble of bioactive lipids, their metabolic enzymes and binding receptors)^18,19^ by microgravity and its involvement in the osteogenic process. The SERiSM experiment was approved by the National Aeronautics and Space Administration (NASA) and was launched to the ISS on board the SpaceX Dragon Spacecraft CRS-12 from Cape Canaveral, Florida (USA), on August 14^th^, 2017, in the frame of the VITA mission of ASI (Fig. 1; for a detailed SERiSM mission profile see Supplementary Figure S1); then, it was incubated at 37 °C inside the ESA KUBIK incubator in the Columbus module onboard ISS. Preliminary data on the modulation of the endocannabinoid system under microgravity have been recently reported^20^. Here, we show that proteomic changes occur in BDSCs upon treatment with rapamycin, speaking in favour of an osteoblastic differentiation of these cells that shows a different timing under microgravity conditions compared to Earth.

It should be recalled that epigenetic regulation is deeply involved in bone homeostasis, and indeed histone modifications are considered the switches used by cells to control differentiation^20–24^. The expression of genes involved in bone remodeling, such as Runx2 and osteocalcin, is accompanied by the increase in methylation and acetylation of Runx2 promoter histones. Here, we also demonstrate that epigenetic modifications occur at H3 histone, and could support osteogenic differentiation of BDSCs on board the ISS.

## Results

### Proteomic changes

**Figure 2:**
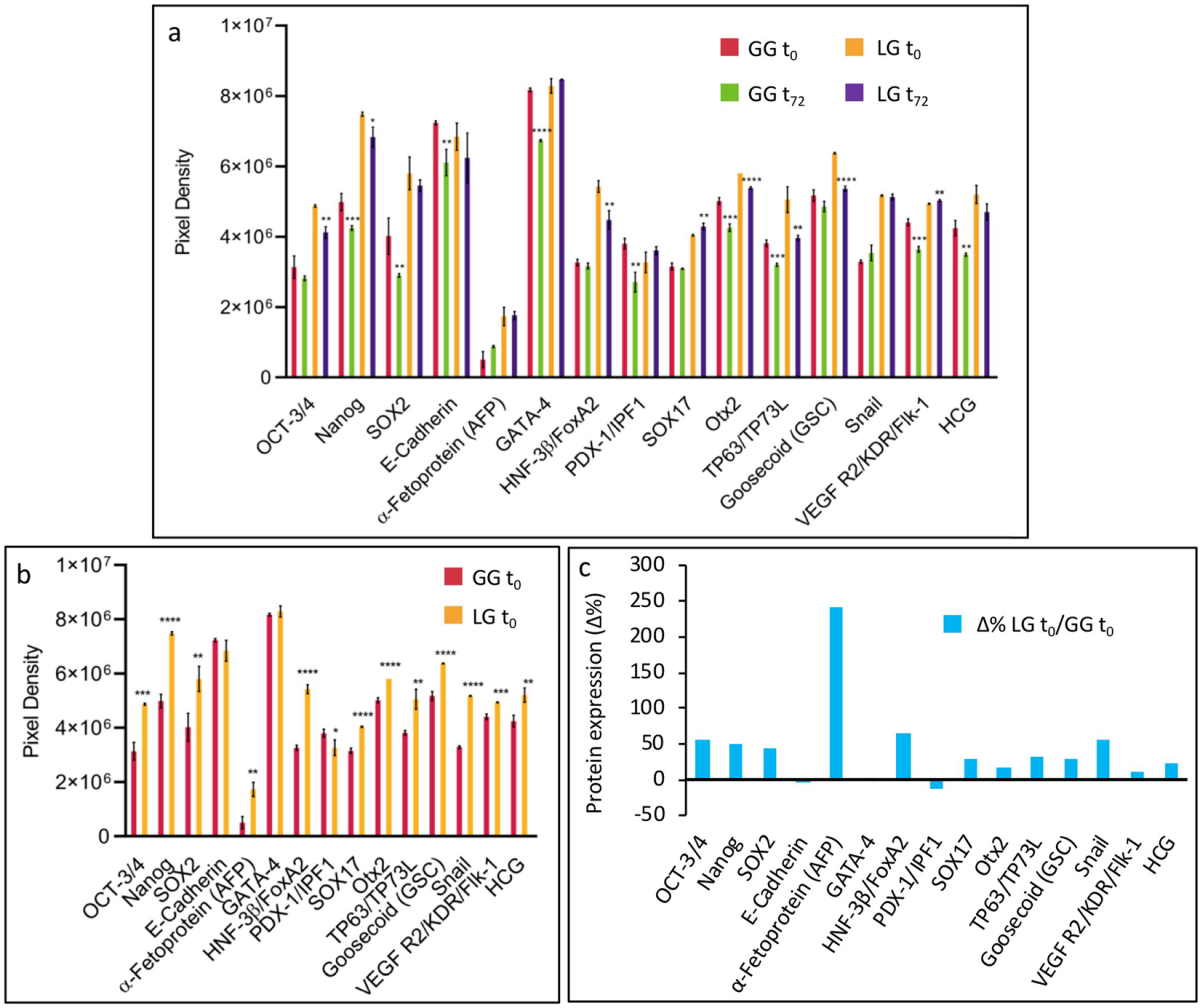
Proteome Profiler Array. (**a**) Human Pluripotent Stem Cell Array results on BDSCs controls (LG t_0_ and GG t_0_) and after 72 hours of differentiation (LG t_72_ and GG t_72_). (**b**) Statistical significance of proteomic profile experiments on BDSCs controls (LG t_0_ and GG t_0_). (**c**) Changes in protein expression measured as Δ% BDSCs LG t_0_/BDSCs GG t_0_. *p < 0.05, 0.0332 < **p < 0.0021, 0.0021 < ***p < 0.0001, ****p < 0.0001.

To study osteogenic differentiation of BDSCs under low gravity conditions (LG) on board the ISS and at ground gravity (GG) on Earth, expression levels of 15 proteins were analyzed as markers of pluripotency, commitment and stem cell differentiation (see Supplementary Table S1 for details). Because of the paucity of the biological material available, limited by the many constraints of Space research under true microgravity conditions, a “Proteome Profiler Array” (Human Pluripotent Stem Cell Array Kit) was used to analyze BDSCs at time zero (LG t_0_ and GG t_0_), and after 72 hours of differentiation (LG t_72_ and GG t_72_), as detailed in Materials and Methods. Thus, we were able to analyze 15 protein markers, some of which are important to determine stem cell phenotype, and others are necessary for stem cell commitment (Supplementary Table S1). The overall data obtained by proteome analysis are shown in Fig. 2a, and are described in detail below.

### Effect of the space flight on BDSC controls

Figure 2b shows densitometric analysis of the data obtained from BDSCs LG t_0_ and GG t_0_ only. In both controls (LG and GG) BDSCs showed a pluripotent stem cell phenotype, as demonstrated by the expression of the transcriptional factors Oct3/4, Sox2 and Nanog. Figure 2c shows that almost all proteins analyzed increased on board the ISS, compared to Earth (BDSCs LG t_0_/GGt_0_). The difference in protein expression between the two controls (LG and GG) led us to normalize the data as Δ% for all timepoints, in order to compare all subsequent results.

### Markers of pluripotency

**Figure 3:**
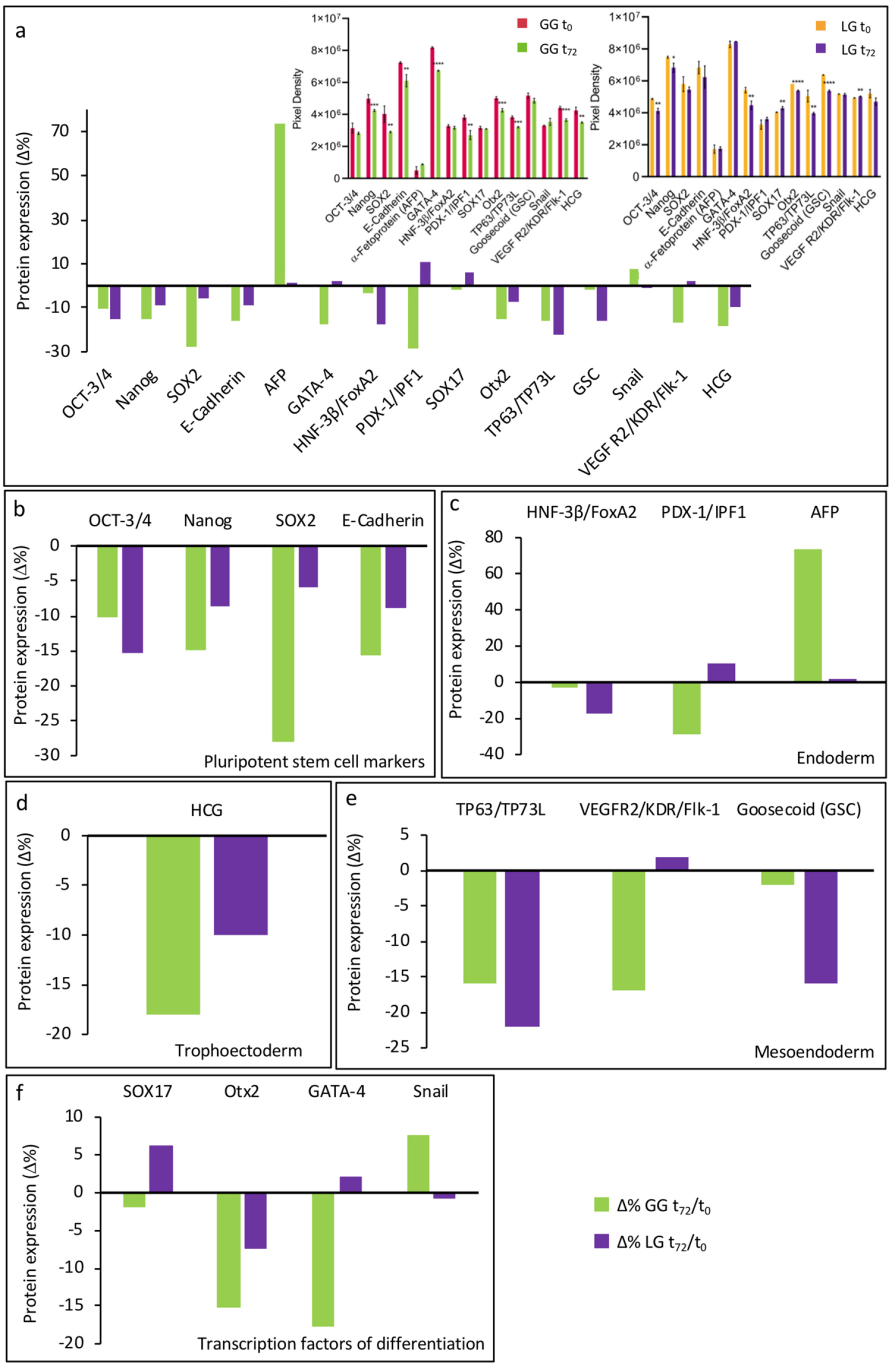
Proteome Profiler Array. (**a)** BDSCs GG and LG proteomic profile comparison after 72 hours of differentiation (Insets in panel a are the graphical statistical significance experiments on BDSCs GG t_72_/t_0_ and BDSCs LG t_72_/t_0_); (**b**–**f)** Changes in protein expression measured as Δ% BDSCs GG t_72_/t_0_ and BDSCs LG t_72_/t_0_ concerning: the decrease of the four pluripotent stem cell markers expression **(b)**; the differences in expression of endoderm **(c)**, trophoectoderm **(d)** and mesoendoderm **(e)** markers, and the differential expression of transcription factors **(f)**. *p < 0.05, 0.0332 < **p < 0.0021, 0.0021 < ***p < 0.0001, ****p < 0.0001.

Figure 3a shows protein expression changes (as Δ%) that occurred after 72 hours of differentiation on ground (GG BDSC t_72_/GG BDSC t_0_) and on board the ISS (LG BDSC t_72_/LG BDSC t_0_). The raw data of densitometric analysis are shown as insets of Fig. 3a, and relative p values are reported in Supplementary Table S2. Figure 3b shows the Δ% in LG BDSCs t_72_ and GG BDSCs t_72_ of the embryonic markers Sox2, Oct3/4, Nanog and E-cadherin, that are responsible for the maintenance of stem cell pluripotency. It was found that Sox2, Nanog and E-cadherin were less expressed in GG BDSCs than in LG BDSCs, while Oct3/4 decreased more markedly in LG BDSCs after 72 hours of osteogenic differentiation.

### Markers of commitment

Figure 3c shows markers of endodermal commitment, such as PDX/IPF1 (insulin promoter factor 1), a transcription factor necessary for pancreatic development, and HNF-3b/FoxA2, that regulates the expression of hepatotrophic factor ALR in liver cells. Of note α-fetoprotein (AFP), a glycoprotein produced in early fetal life by the liver, was the only marker that did not change its expression in LG BDSCs t_72_ when compared to its LG BDSCs t_0_ control. In Fig. 3d, a marker of the trophoectoderm is shown: HCG (human chorionic gonadotropin), a glycoproteic hormone secreted during pregnancy by embryonic trophoblast cells. Also in this case, a difference between LG and GG BDSCs was observed after 72 hours of differentiation, along with a decreased expression on ground.

In Fig. 3e markers of mesoendodermal germinal layer are shown, namely: TP63/TP73L, a key player in embryonic urogenital development; Goosecoid (GCS), a homeobox protein expressed in cells that become pharyngeal endoderm, head mesoderm and notochord; and VEGF R2/KDR/Flk-1, that plays critical roles in skeletal development and directly controls differentiation and function of osteoblasts. Only VEGF R2/KDR/Flk-1 expression was found to increase in LG BDSCs t_72_, as expected during an osteogenic differentiation process.

### Markers for transcriptional processes

Figure 3f highlights the changes in the expression of 4 transcription factors that are critical in determining whether the differentiation process can take place. Otx2 and Snail are negative transcriptional factors, necessary for the maintenance of the stemness phenotype, while GATA4 and SOX17 are considered fundamental in promoting differentiation, in particular towards osteogenesis. On board the ISS, BDSCs showed a remarkable increase of SOX17 and GATA4 after 72 hours, and a more pronounced expression of Otx2 and Snail when compared with the ground control cells.

### Epigenetic modifications

**Figure 4:**
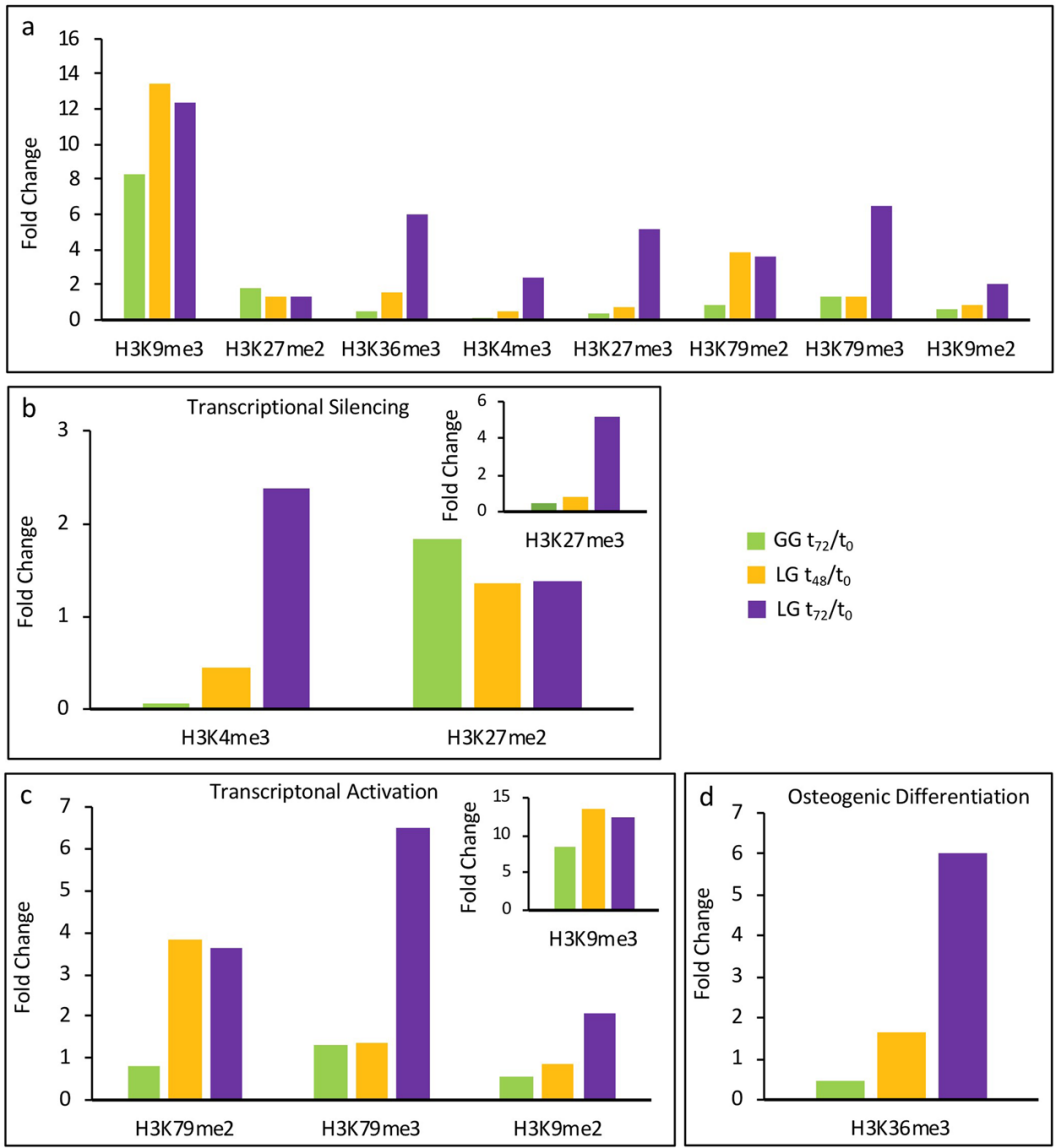
Epigenetic H3 modifications during differentiation in LG and GG conditions (expressed as fold changes). (**a)** Global histone modifications occurred, respect to controls, at 48 (LG) and 72 hours (GG, LG) of osteogenic differentiation (**b**) Histone modifications concerning the gene silencing occurred during the differentiation process. (**c)** Histone modifications concerning the gene opening and chromatin remodeling during differentiation. (**d**) Histone modification related to osteogenic differentiation.

Figure 4a shows the results on 8 epigenetic modifications of H3 histone (expressed as fold changes) in BDSCs under GG and LG conditions at t_0_, t_48_ and t_72_. Due to the paucity of the biological material, we focused on the following lysine di/tri-methylations: H3K79me2/me3, H3K27me2/me3, H3K4me3, H3K9me2/me3 and HK36me3. Indeed, these epigenetic modifications are known to be involved in: heterochromatization of DNA and transcriptional silencing (H3K27me2, H3K27me3 and H3K4me3, shown in Fig. 4b); chromatin remodeling and transcriptional activation during differentiation (H3K79me2, H3K79me3, H3K9me2 and H3K9me3, shown in Fig. 4c); and more directly osteogenic differentiation (HK36me3, Fig. 4d). Similar changes were found at LG and at GG, yet to different extents. An increased trimethylation of lysine 4 (H3K4me3) and di/tri-methylation of lysine 27 (H3K27me2/3) were observed in LG BDSCs (both t_48_ and t_72_), compared to GG BDSCs at t_72_; at the same timepoints di/tri-methylation of lysine 79 (H3K79me2/3) was also increased. Additionally, we found very high levels of di/tri-methylation of H3K9 in both LG and GG cells, while in LG BDSCs (t_48_ and t_72_) we observed an increased trimethylation of H3K36 compared to GG BDSCs.

## Discussion

Proteomic and epigenetic changes occurring in BDSCs under true low gravity conditions on board the ISS were interrogated in a model of osteogenic differentiation that had been previously validated on Earth^10^. The advantage of an easy accessibility of blood-derived stem cells, compared to other (e.g., mesenchymal) stem cells, makes them a promising tool to identify biomarkers and/or therapeutic targets potentially useful for monitoring and/or treating osteoporosis in Space^11–15^. Of note, our preliminary data on Earth demonstrated that already after 72 hours of treatment with rapamycin and a suitable scaffold, BDSCs differentiated with a mesoendodermal commitment, secreting calcium phosphate and forming hydroxyapatite^10^. Therefore, we chose to perform proteomic analysis at t_0_ and t_72_, also including a t_48_ timepoint to interrogate possible early changes in epigenetic regulation of gene expression. Protein expression analysis demonstrated changes in LG t_72_ samples when compared to the GG t_72_ controls. Almost all proteins analyzed increased on board the ISS compared to Earth (LG BDSCs t_0_ compared to GG BDSCs t_0_), suggesting that the flight towards the ISS affected *per se* gene expression. Thus, we chose to normalize the data in order to properly compare ISS and Earth samples.

In LG and GG samples at t_72_, a decrease in the expression of the 4 embryonic markers Sox2, Oct3/4, Nanog and E-cadherin was observed (Fig. 3b), suggesting that BDSCs were losing their pluripotency both on board the ISS and on Earth. Yet, under microgravity the process was more pronounced. Differences between LG and GG samples were more evident when endodermal, trophoectodermal and, more notably, mesoendodermal markers were analyzed (Fig. 3c,d,e). In particular, after 72 hours only LG cells showed an increase of VEGFR-2, that is fundamental to recruit osteoprogenitor cells and thus form bones^25^. In keeping with these data, Dhaliwal and colleagues have recently demonstrated enhanced osteogenesis in bone marrow-derived human mesenchymal stem cells through induction of VEGFR-2^26^.

Taken together, these data suggested an earlier activation of the differentiation processes in LG *versus* GG cells, an hypothesis that was confirmed by comparing the expression of the 4 transcription factors Otx2, Snail, GATA4 and Sox17. While Otx2 decreased in both LG and GG cells, Snail decreased in LG cells and increased in GG cells; both Sox17 and GATA4 increased in LG cells and showed little expression in GG cells at t_72_ compared to t_0_ (Fig. 3f). Also the decrease of Otx, which counteracts commitment and promotes pluripotency^27,28^, supported our findings. The data on Sox17 appeared of particular interest, because Sox17 is a transcriptional regulator that promotes differentiation of pluripotent cells; indeed, Sox17-deficient embryonic stem cells do not differentiate into extraembryonic cells and keep on expressing pluripotency-associated transcription factors like Oct4, Nanog and Sox2. Instead, forced expression of Sox17 downregulates embryonic stem cell-associated gene expression, and activates genes responsible for differentiation^29^. Thus, low expression of Sox17 in GG BDSCs after 72 h of induction suggests that these cells are still at the beginning of the differentiation process, while Sox17 increase in LG BDSCs suggests that these cells are at a later stage of differentiation.

GATA4 is consistently expressed in pre-osteoblast cells and gradually down-regulated during osteoblast differentiation^30^. Guo and colleagues recently suggested a role for GATA4 in maintaining normal trabecular bone mass. Interestingly, both *in vivo* and *in vitro* reduction of GATA4 correlates with reduced Runx2 gene expression, along with reduced osteoblast mineralization^31^. It should be recalled that Runx2 is a transcription factor crucial for osteoblast differentiation^32^, as it is responsible for the synthesis of osteoblastic proteins like Osterix (Otx) and osteocalcin (Ocn)^33–35^. It has been suggested that GATA4 binds near the Runx2 promoter and enhancer, and helps maintaining open chromatin to regulate Runx2 expression that leads to bone mineralization^31^. In the same context, also Snail plays a crucial role in osteogenic differentiation by acting as a direct Runx2 repressor^36^. Thus, GATA4 and Snail have opposite roles in osteogenic differentiation by enhancing or repressing Runx2, respectively^37,38^. Taken together, in LG BDSCs the increase of GATA4 and Sox17 combined with the decrease of Snail and the increase of VEGFR-2 leads to activation of Runx2, and hence of osteogenesis.

In order to ascertain whether regulation of gene expression could depend on epigenetic mechanisms, we interrogated changes of histone H3 that is crucial in this type of gene control^39^. Indeed, during the differentiation process cells become more mature, lose pluripotency and undergo epigenetic rearrangements that shut off stemness genes, making cells resistant to returning to the undifferentiated state^39^. Figure 4a summarizes the overall modifications observed at t_72_ and at the earlier point t_48_. During stem cell differentiation, H3K4me3, H3K27me2/3, H3K79me2/3 and H3K9me2/3 residues are engaged in cellular reprogramming that controls gene expression^39^. We found that these changes did occur in LG cells (Fig. 4b and c, respectively).

The presence of di/tri-methylated H3K4 and H3K27 generally indicates inhibition of transcription and heterochromatinization, while during cellular differentiation their modulation is indicative of gene opening and closing^39^. Our results are consistent with literature data, reporting an increase in H3K27me3 and H3K4me3 at the beginning of differentiation, when chromatin is reassembled to silence genes responsible for stemness and to open lineage-specific genes only^40^.

The methylation of H3K79 (me2/3) seems also remarkable, because it is considered to impair cell reprogramming^41^. Indeed, it has been shown that a high level of H3K79 di/tri-methylation prevents reprogramming of an adult cell towards iPSC, while its reduction enhances the number of reprogrammed cells^41,42^.

Another epigenetic modification considered an obstacle to cellular reprogramming is the methylation of H3K9 (me2/3)^43^. H3K9me3 prevents initial binding of OSKM (Oct3/4, Sox2, Klf4 and c-Myc), thus hindering iPSC formation and reducing reprogramming efficiency^44^. We observed a high increase of H3K9me2/3, suggesting that BDSCs are leaving the pluripotent state to enter the differentiation program. Once again, it appears that the differentiation process builds up epigenetic barriers to drive cell fate, while stem cells do not show relevant histone modifications such as H3K79me2/3 and H3K9me2/3^45–48^.

Our present results are in agreement with those by Dhaliwal and colleagues^24^, who demonstrated that epigenetic modifications like H3K9me3, H3K27me3 and H3K4me3 can be modulated to direct optimal osteoblastic differentiation within 72 hours, in the presence of osteogenic cues, and can regulate osteogenic differentiation of mesenchymal stem cells. An additional modification that we observed was trimethylation of H3K36 (Fig. 4d), that is known to increase during osteogenic differentiation^49–51^. H3K36me3 is required in promoters for Osterix, a specific transcription factor of osteoblasts^52^. Consistently, it increased both in LG and in GG BDSCs, confirming that osteogenic differentiation was activated. The different extent of epigenetic changes in LG *versus* GG BDSCs at t_72_ may be a corollary to the differences in protein expression observed at the same timepoint, indicating that these cells follow the same trends during their differentiation, but with an earlier start under LG conditions.

**Figure 5:**
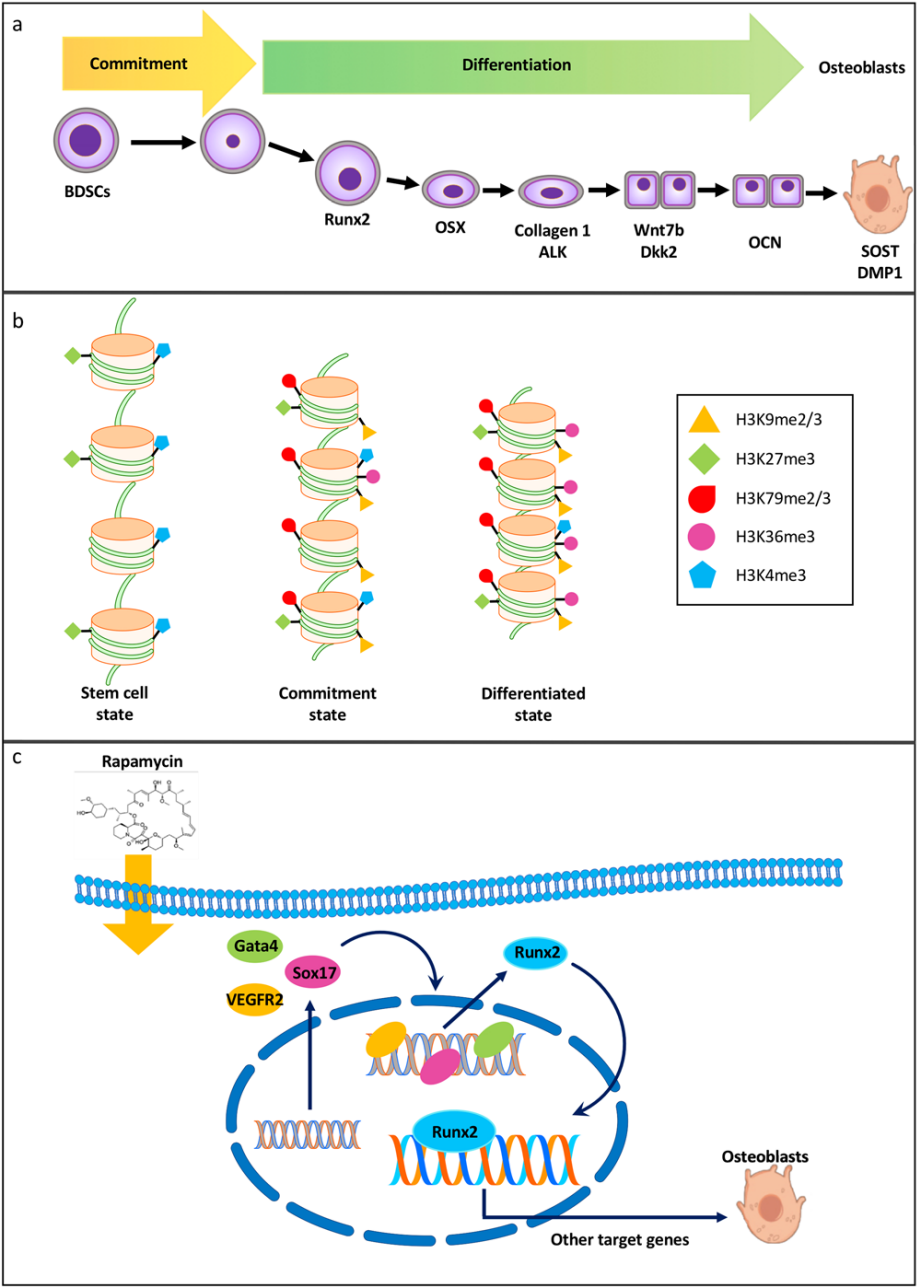
From stem cells to osteoblasts: proteomic and epigenetic pathways. (**a**) Timeline of protein expression during osteoblastic differentiation. (**b)** Proposed epigenetic changes occurring during stem cell differentiation. (**c)** Proposed role of transcription factors and VEGFR2 during the first steps of osteogenesis differentiation.

In conclusion, osteogenic differentiation of BDSCs induced by rapamycin follows a coordinated pattern in protein expression and epigenetic arrangement, as schematically depicted in Fig. 5. GG BDSCs at t_72_ are still in an osteogenic commitment phase, while the early expression of GATA-4 and Sox17 in LG BDSCs at t_72_ indicates that under microgravity conditions these cells are already in full osteogenic differentiation, rather than simply committed (Fig. 5a). Moreover, the results obtained in this investigation are in agreement with literature data showing how genes involved in embryonic stem cell maintenance exhibit high levels of H3K27me3 and H3K9me3, leading to a stable chromatin^53^. In addition, H3K36me3 must increase during osteogenic differentiation^49^, as we indeed observed in our cells (Fig. 5b). Overall, we suggest that rapamycin induces transcriptional activation of BDSCs through increased GATA4 and Sox17 that modulate Runx2 expression by means of VEGFR-2. In turn, Runx2 induces osteogenic differentiation by activating the transcription of Osterix (Osx) and Osteocalcin (Ocn) (Fig. 5c). Additional studies are warranted to ascertain the possible exploitation of these data to identify new biomarkers and therapeutic targets to treat osteoporosis, not only in Space but also on Earth.

## Materials and Methods

### Preparation of BDSCs

Human BDSCs were purified from blood samples, as previously reported^12^. Briefly, the nucleated blood cell fraction was isolated by ammonium chloride incubation (dilution 1: 3 in NH_4_Cl 1 M), centrifuged at 1023 g for 20 minutes and washed several times with phosphate-buffered saline (PBS), pH7.2 (Base Catalog BR0014G, Oxoid, Hampshire, England), to remove the majority of erythrocytes. Cells were then resuspended in 5 ml PBS and incubated for 72 h at 37 °C in the presence of 50 nM macrophage colony-stimulating factor (Base catalog M9170, Sigma-Aldrich, St. Louis, MO), and 5 μM gentamicin sulphate (Base catalog L0012, BioWest, Nuaillé, France). For the VITA mission experiments, 16 samples were prepared and distributed as follows: 6 BDSC controls (LG t_0_), 4 samples for the 48 hours BDSCs (LG t_48_), 6 samples for the 72 hours BDSCs (LG t_72_). In the proteomic and epigenetic experiments 3 LG t_0_, 2 LG t_48_ and 4 LG t_72_ were used. Healthy blood donors gave their written informed consent to the study according to the Legislative Decree n. 196/2003. The study was approved by the Ethical Committee of Campus Bio-Medico University of Rome and was conducted according to the ethical principles arising from Helsinki Declaration. Participation of the subjects was voluntary and unpaid.

### SERiSM Hardware

Cell growth and differentiation of BDSCs occurred in the hardware developed on purpose by Kayser Italia Srl, shown in Supplementary Figure S2.

The SERiSM Experiment Unit (KEU-RO) is a device capable of performing automatic cell culture of non–adherent cells in microgravity. It is equipped with reservoirs for chemicals and a culture chamber allowing cell growth in suspension. The scientific protocol is led by the KEU-RO electronics following a predefined timeline. Each KEU-RO Experiment Unit (EU) is made of a semi-crystalline thermoplastic polymer with excellent mechanical and chemical resistance properties, biologically inert. Cross contamination among the fluids chambers are avoided due to proper sealing gaskets. The EU itself provides one *Level of Containment* (LoC) that is increased to two by using KIC-SL container class. The experiment is fully autonomous; all the actions are electrically controlled by a predefined timeline uploaded into the on-board microcontroller. Housekeeping data are recorded during the mission and downloaded at re-entry.

The typical fluidic concept carries out the KEU-RO experimental protocol which relies on three main steps, i.e. Activation, Incubation, Fixation. On the whole, the actions performed by the fluidic system are led by preloaded spring actuators activated by the control electronics. Such mechanism pushes the pistons inward displacing the fluids (Activator or Fixative) contained into the chemicals reservoirs (Activator or Fixative reservoir) towards the Culture Chamber (CC). Short channels connect independently the reservoirs to the CCs so that cells are activated or fixed. The SERiSM Experiment Unit (KEU-RO) assembled with control electronics was integrated inside the KIC-SL containers (Kayser Italia Containers- Single Level), and then placed inside the BIOKON transportation Container for the upload onboard launcher. Onboard ISS the SERiSM Experiment Hardware was inserted inside the ESA KUBIK incubator in the ISS Columbus module, set at 37 °C.

### Osteogenic differentiation

To promote *in vitro* osteogenic differentiation, BDSCs were grown in DMEM-F12 (Lonza, Belgium, BE12-719F) supplemented by 10% FBS (not-USA origin, Sigma, USA, F9665), 1% penicillin (100 units/ml)/streptomycin (100 mg/ml) (Lonza, Belgium, DE17-602E), rapamycin 10 nM as osteogenic inductor (Sigma-Aldrich, St. Louis, MO, R0395) and Bio-Oss scaffold (Geistlich, Switzerland, 30643.3/500079), as previously reported^10,15^.

### Proteome profiler

Changes in protein expression were evaluated by a Proteome Profiler Array (Human Pluripotent Stem Cell Array Kit, R&D Systems, Abingdon, UK, ARY010), according to the manufacturer’s instructions. For the proteomic experiments, two samples were used for each timepoint, each in duplicate (BDSCs LG t_0_, BDSCs LG t_72_, BDSCs GG t_0_, BDSCs GG t_72_). Briefly, cells were solubilized in 100 μl lysis Buffer supplemented with protease inhibitor cocktail (Calbiochem, EMD Millipore, Billerica, MA USA, 535140-1 ML), centrifugated a 4 °C at 14000 *g* for 5 minutes and the supernatant collected. Protein concentrations were assessed by Bradford assay. For each sample 200 μg of proteins were used to perform the array. After development of autoradiographies, pixel density of each marker was evaluated using ImageJ analysis software and Microsoft Office and the results expressed in Relative-delta percentage as suggested by manufacter’s protocol:

$${\rm{Relative}}\,{\rm{\Delta }}( \% )=[\frac{(Pixel\,Density\,of\,sample2-Pixel\,Density\,of\,sample1)}{Pixel\,Density\,of\,sample1}]\times 100$$

### Total histone purification

Histones were isolated using EpiQuik Total Histone Extraction Kit (Epigentek, Farmingdale, NY, #OP-0006) according to the manufacturer’s instructions. For epigenetic experiments one sample of BDSCs GG t_0_, two samples of BDSCs GG t_72_, one sample of BDSCs LG t_0_, two samples of BDSCs LGt_48_, two samples of BDSCs LG t_72_ were used. Briefly, the cellular pellet was resuspended in 1 ml Diluted Pre-Lysis Buffer (1X) and was placed on ice for 10 minutes under stirring to remove the plasmatic membranes. The lysate was centrifuged at 9391 *g* for 1 minute at 4 °C and the supernatant removed. The pellet was resuspended in 50 μl of the Lysis Buffer and incubated on ice for 30 minutes. The lysate was centrifuged at 13523 g for 5 minutes at 4 °C to remove the nuclear membranes and the supernatant transferred to a new tube. For each sample, 0.3 volumes of Balance Buffer were immediately added to the supernatant without DTT since its presence causes scaffold precipitates that hindered histones extraction. Histones concentration was assessed by Bradford assay.

### Histone H3 modification

To evaluate H3 modifications the EpiQuik Histone H3 Modification Multiplex Assay Kit (Epigentek, Farmingdale, NY, #P-3100) was used according to the manufacturer’s instructions. Due to the limited amount of material available, the following histone modifications were considered for each sample: H3K4me3, H3K9me2, H3K9me3, H3K27me2, H3K27me3, H3K36me3, H3K79me2 and H3K79me3. The amount of each histone modification was compared to the own total histone and then expressed as a percentage, using the following formulas, as described in the manufacturer’s protocol.

$${\rm{H}}3\,{\rm{Modification}}\,{\rm{or}}\,{\rm{Total}}\,{\rm{H}}3({\rm{ng}}/\mu g\,{\rm{protein}})=[\frac{(Sample\,OD-Blank\,OD)/S}{(Assay\,Control\,OD-Blank\,OD)/P}]\times 1000$$

$${\rm{H}}3\,{\rm{Modification}} \% =[\frac{Amount\,of\,H3\,modification(\frac{ng}{\mu g}protein)}{Amount\,of\,total\,H3(\frac{ng}{\mu g}protein)}]\times 100$$

To better highlight the results obtained, we reported each H3 modification as fold change, using the following formula:

$$Fold\,Change=[\frac{H3\,modification\, \% \,sample2-H3\,Modification\, \% \,sample1}{H3\,Modification\, \% \,sample1}]$$

### Statistical analysis

Statistical analysis of proteomics data was performed with GraphPad Prism3 by multiple T test (one per line). Statistical significance was determined by using the Holm-Sidak method, with alpha = 0.05. Each row was analyzed individually. The test number was equal to 15. p < 0.05 (*), 0.0332 < p < 0.0021 (**), 0.0021 < p < 0.0001 (***), and p < 0.0001 (****), as reported in Supplementary Table S2.

## Supplementary information

Supplementary Data
