# Supplementary material for: Human osteogenic differentiation in Space: proteomic and epigenetic clues to better understand osteoporosis
Source: Sci Rep. 2019 Jun 6;9:8343. doi: 10.1038/s41598-019-44593-6 (PMC6554341; doi:10.1038/s41598-019-44593-6)
Supplement: Supplementary file 1 — Supplementary Data [file 41598_2019_44593_MOESM1_ESM.pdf]

Human osteogenic differentiation in Space: proteomic and epigenetic clues to better understand osteoporosis

Gambacurta Alessandra[1,2,#], Merlini Giulia[1], Ruggiero Cristina[1], Diedenhofen Giacomo[1], Battista Natalia[3], Bari Monica[1], Balsamo Michele[4], Piccirillo Sara[5], Valentini Giovanni[5], Mascetti Gabriele[5] and Maccarrone Mauro[6,7, #]

[1]Department of Experimental Medicine, Tor Vergata University of Rome, Rome, Italy
[2]NAST Centre for Nanoscience, Tor Vergata University of Rome, Rome, Italy
[3]Faculty of Bioscience and Technology for Food, Agriculture and Environment, University of Teramo, Teramo, Italy;
[4]Kayser Italia S.r.l., Livorno, Italy
[5]Italian Space Agency, Rome, Italy
[6]Department of Medicine, Campus Bio-Medico University of Rome, Rome, Italy
[7]European Center for Brain Research, IRCCS Santa Lucia Foundation, Rome, Italy

[#]Corresponding authors: A. Gambacurta (gambacur@uniroma2.it), M. Maccarrone (m.maccarrone@unicampus.it).

Supplementary Data

Figure S1

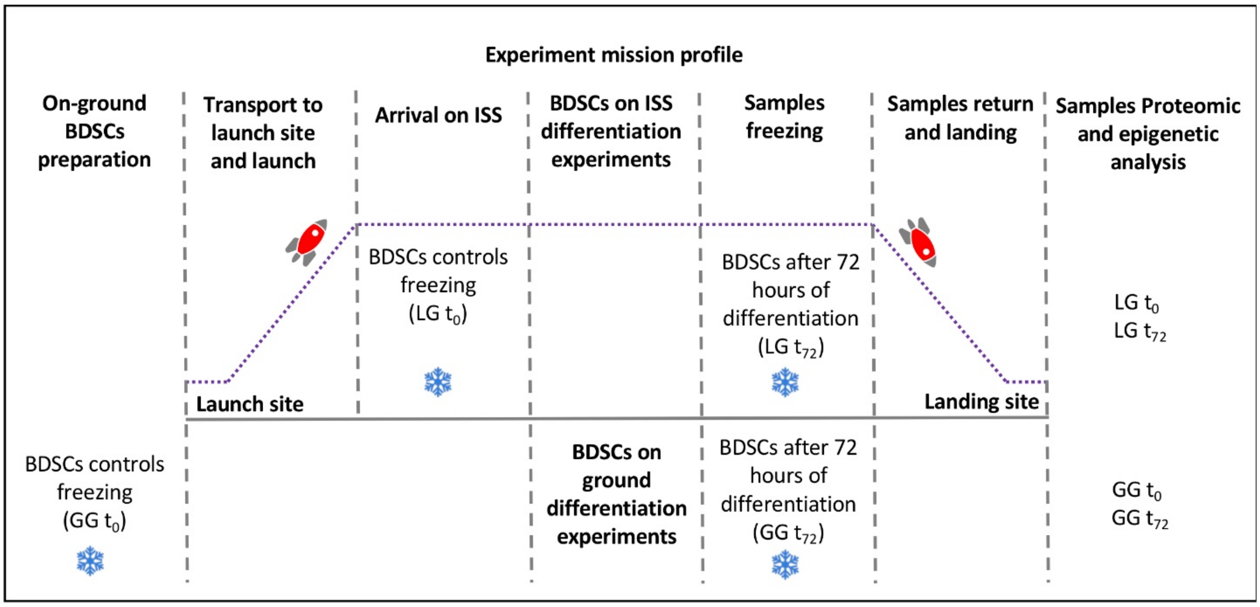

Figure S1. Graphical abstract of the SERiSM mission profile, with timing of all experimental phases. Launch date August 14th 2017, landing date September 27th 2017. ❄ : samples freezing.

Figure S2

a)

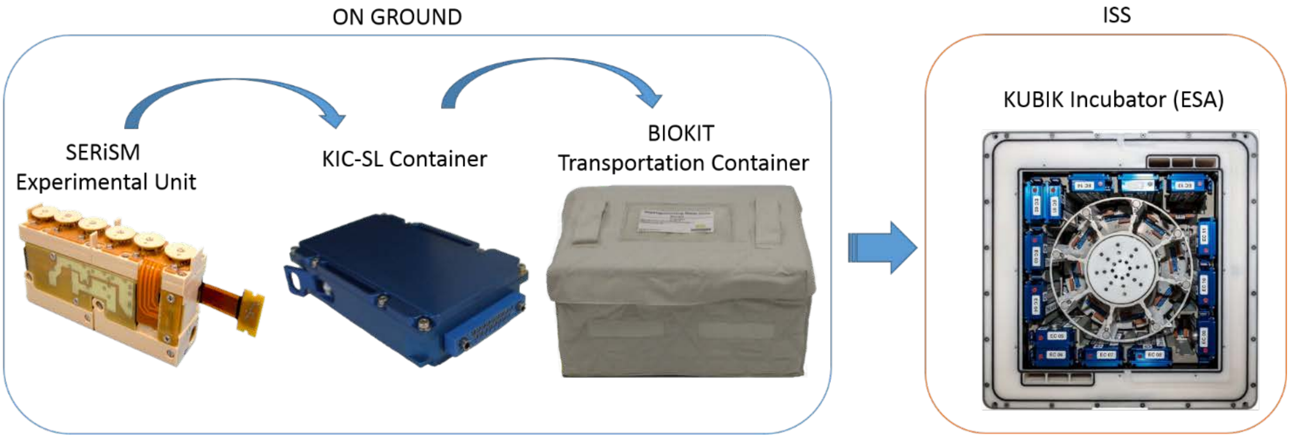

b)

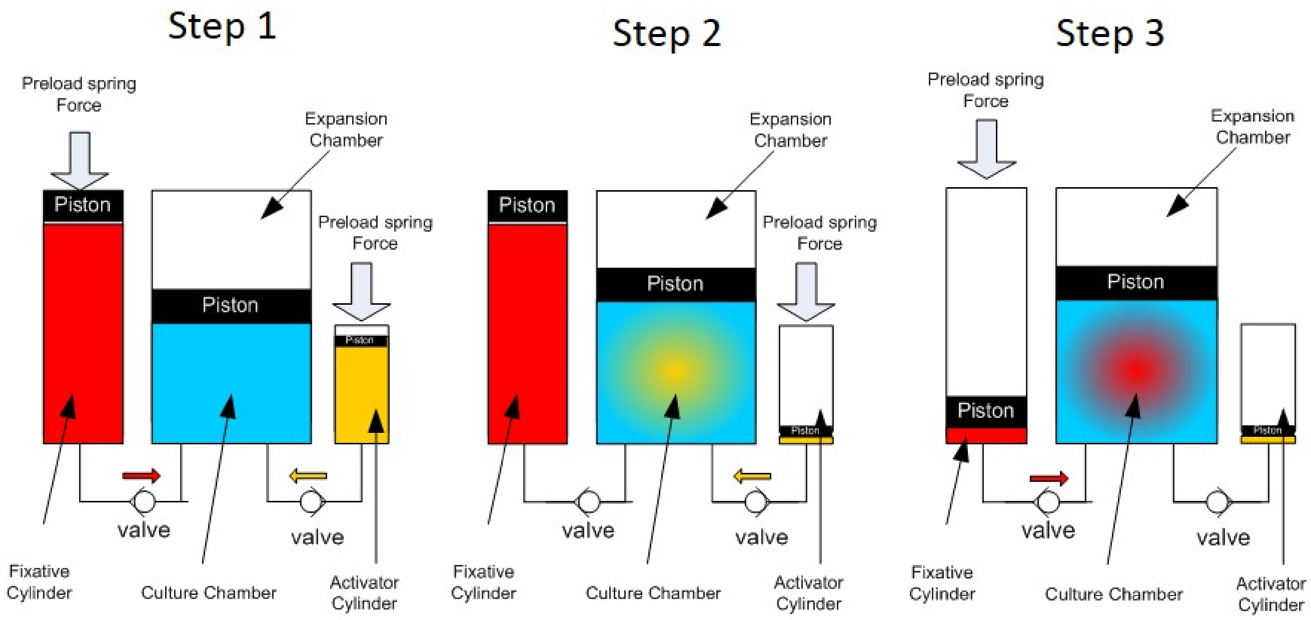

Figure S2. a) Diagram showing the SERiSM experiment hardware: the SERiSM Experiment Unit (KEU-RO) assembled with control electronics is integrated inside the KIC-SL containers (Kayser Italia Containers-Single Level) and placed inside the BIOKON transportation Container for the upload onboard launcher. Onboard ISS the SERiSM Experiment Hardware was inserted inside the ESA KUBIK incubator in the ISS Columbus module, set at 37°C. b) Fluidic path and sequence of activations of the SERiSM Experiment Unit.

Table S1	Stem cell markers		
**OCT-3/4**	Pluripotency marker	**Sox17**	Mesodermal marker [29]
**Nanog**	Pluripotency marker	**Otx2**	Metastable state marker [27, 28]
**SOX2**	Pluripotency marker	**TP63/TP73L**	Mesoendodermal marker
**E-Cadherin**	Pluripotency marker	**Goosecoid (GSC)**	Mesoendodermal marker
**α-Fetoprotein (AFP)**	Endodermal marker	**Snail**	Runx2 repressor [36, 38]
**GATA-4**	Transcription factor [30, 31, 37]	**VEGF R2/KDR/Flk1**	Mesoderm commitment [25, 26]
**HNF-3β/FoxA2**	Endodermal marker	**HCG**	Trophoblast marker
**PDX-1/IPF1**	Pancreatic marker		

Table S1. Protein markers analyzed by the Human Pluripotent Stem Cell Array.

Table S2	Adjusted P Value GG t72/GG t0	Adjusted P Value LG t72/LG t0	Adjusted P Value LG t0/GG t0
**OCT-3/4**	0,2738	0,0015	0,0003
**Nanog**	0,0093	0,0329	<0,0001
**SOX2**	0,0337	0,5581	0,0086
**E-Cadherin**	0,0093	0,5581	0,1729
**α-Fetoprotein (AFP)**	0,1078	0,8515	0,0029
**GATA-4**	<0,0001	0,5141	0,3243
**HNF-3β/FoxA2**	0,2855	0,0108	<0,0001
**PDX-1/IPF1**	0,005	0,3489	0,0481
**SOX17**	0,2855	0,0212	<0,0001
**Otx2**	0,0005	<0,0001	<0,0001
**TP63/TP73L**	0,0002	0,0116	0,0036
**Goosecoid (GSC)**	0,1375	<0,0001	<0,0001
**Snail**	0,2588	0,6234	<0,0001
**VEGF R2/KDR/Flk-1**	0,0003	0,0116	0,0004
**HCG**	0,005	0,177	0,0061

Table S2. Relative p values for each of the 15 markers analyzed by the Proteome Profile Array.
